# Gene expression profiling of primary male breast cancers reveals two unique subgroups and identifies *N-*acetyltransferase-1 (*NAT1*) as a novel prognostic biomarker

**DOI:** 10.1186/bcr3116

**Published:** 2012-02-14

**Authors:** Ida Johansson, Cecilia Nilsson, Pontus Berglund, Martin Lauss, Markus Ringnér, Håkan Olsson, Lena Luts, Edith Sim, Sten Thorstensson, Marie-Louise Fjällskog, Ingrid Hedenfalk

**Affiliations:** 1 Department of Oncology, Clinical Sciences, Lund University, Barngatan 2B, SE 22185 Lund, Sweden; 2 CREATE Health Strategic Center for Translational Cancer Research, Lund University, BMC C13, SE 22184 Lund, Sweden; 3 Center for Clinical Research, Central Hospital of Västerås, SE 72189 Västerås, Sweden; 4 Department of Oncology, Uppsala University, SE 75185 Uppsala, Sweden; 5 Department of Pathology, Lund University Hospital, SE 22185 Lund, Sweden; 6 Department of Pharmacology, University of Oxford, Mansfield Road, Oxford, OX1 3SZ, UK; 7 Department of Pathology, Linköping University Hospital, SE 58185 Linköping, Sweden

## Abstract

**Introduction:**

Male breast cancer (MBC) is a rare and inadequately characterized disease. The aim of the present study was to characterize MBC tumors transcriptionally, to classify them into comprehensive subgroups, and to compare them with female breast cancer (FBC).

**Methods:**

A total of 66 clinicopathologically well-annotated fresh frozen MBC tumors were analyzed using Illumina Human HT-12 bead arrays, and a tissue microarray with 220 MBC tumors was constructed for validation using immunohistochemistry. Two external gene expression datasets were used for comparison purposes: 37 MBCs and 359 FBCs.

**Results:**

Using an unsupervised approach, we classified the MBC tumors into two subgroups, luminal M1 and luminal M2, respectively, with differences in tumor biological features and outcome, and which differed from the intrinsic subgroups described in FBC. The two subgroups were recapitulated in the external MBC dataset. Luminal M2 tumors were characterized by high expression of immune response genes and genes associated with estrogen receptor (ER) signaling. Luminal M1 tumors, on the other hand, despite being ER positive by immunohistochemistry showed a lower correlation to genes associated with ER signaling and displayed a more aggressive phenotype and worse prognosis. Validation of two of the most differentially expressed genes, class 1 human leukocyte antigen (*HLA*) and the metabolizing gene *N*-acetyltransferase-1 (*NAT1*), respectively, revealed significantly better survival associated with high expression of both markers (HLA, hazard ratio (HR) 3.6, *P *= 0.002; NAT1, HR 2.5, *P *= 0.033). Importantly, NAT1 remained significant in a multivariate analysis (HR 2.8, *P *= 0.040) and may thus be a novel prognostic marker in MBC.

**Conclusions:**

We have detected two unique and stable subgroups of MBC with differences in tumor biological features and outcome. They differ from the widely acknowledged intrinsic subgroups of FBC. As such, they may constitute two novel subgroups of breast cancer, occurring exclusively in men, and which may consequently require novel treatment approaches. Finally, we identified NAT1 as a possible prognostic biomarker for MBC, as suggested by NAT1 positivity corresponding to better outcome.

## Introduction

Male breast cancer (MBC) is a rare cancer form accounting for only 0.6% of all breast cancer cases in the Nordic countries [1]. MBC is similar to female breast cancer (FBC) in many ways and is often likened to post-menopausal breast cancer in women due to the high prevalence of estrogen receptor (ER) positivity and relatively high age at onset. There are nevertheless also distinct differences; there is an ongoing debate regarding the level of similarity between FBC and MBC, and whether MBC may be a unique tumor type with biological features and clinicopathological parameters distinct from FBC [2-4]. MBC tumors are more frequently hormone receptor positive than FBC tumors (ER positivity 91% *vs*. 76% and progesterone receptor (PR) positivity 81% *vs*. 67%, respectively). Human epidermal growth factor receptor 2 (HER2) over-expression and/or amplification appear less frequent in MBC and the mean age at diagnosis is approximately five years older than for women [2,3,5,6]. Risk factors include hormonal imbalances (for example, caused by liver disease, Klinefelter's syndrome or obesity), genetic predisposition (mainly due to *BRCA2 *mutations) and environmental factors (for example, exposure to chronic heat or radiation) [7,8]. Survival rates have been debated, with some studies finding that men diagnosed with breast cancer have a worse prognosis than women [9,10], whereas other studies have reported similar prognoses [11,12]. The rarity of the disease has, however, precluded randomized trials for optimizing patient management; thus, recommendations for treating MBC are extrapolated from small retrospective trials and prior knowledge of FBC [13].

Importantly, no major progress has been made in the treatment of MBC since the introduction of hormonal therapy; survival rates have not improved over the last decades, unlike the female counterpart. Refined, comprehensive classification and identification of novel biomarkers will greatly increase our understanding of the pathobiology of the disease, and enable personalized clinical management as well as rationales for targeted therapy. We have previously described two genomic subgroups of MBC by array-based comparative genomic hybridization (aCGH) [14], and a few smaller studies have described specific differences between MBC and FBC based on gene expression (GEX) [15], microRNA [16,17] and genomic profiles [14,18], respectively.

In the present study, we aimed to understand MBC on the transcriptional level and to subclassify tumors into comprehensive subgroups. We also wanted to further validate the previously identified genomic subgroups that were based on the same cases [14], and to compare MBC with FBC. To this end, molecular profiling has been extensively applied to FBC by numerous independent researchers, resulting in the subdivision into gene expression-based 'intrinsic' subgroups associated with differences in survival as well as biological phenotypes [19-22]. Herein, we describe two stable subgroups of MBC, luminal M1 and luminal M2, respectively, highly correlated to the recently described MBC genomic subgroups [14]. Remarkably, these subgroups were distinct from the well-established intrinsic subgroups of FBC, and may, as such, represent unique subtypes of breast cancer arising exclusively in males. The largest subgroup (luminal M1), comprising two-thirds of all cases, displayed a more aggressive phenotype and worse prognosis compared to the other cases, while high expression of immune response and ER-related genes was seen in the smaller subgroup (luminal M2). Finally, we identified *N*-acetyltransferase-1 (NAT1) as a potential prognostic biomarker in MBC.

## Materials and methods

### Tumor tissue

All cases of MBC diagnosed between 1983 and 2009 in the Lund and Uppsala-Örebro regions with sufficient tumor material available were identified. Fresh frozen and paraffin-embedded primary tumors were obtained from the Southern Sweden Breast Cancer Group's tissue bank at the Department of Oncology, Skåne University Hospital, Uppsala University Hospital and Örebro Hospital. A physician (CN) reviewed all patient charts and collected clinicopathological data. A pathologist (ST) graded all tumors to current pathological standard; all histological grades were represented. ER, PR and HER2 were re-evaluated (see [6] for further details). The patients had received different combinations of adjuvant treatment, including hormonal, chemotherapy and radiation treatment, and the mean follow-up time was 4.6 years (range 0.04 to 15 years). The mean age at diagnosis was 70 years (range 23 to 98). Five known *BRCA2 *mutation carriers, but no known *BRCA1 *mutation carriers, were included; however, most of the patients were not screened for *BRCA1/2 *mutations. The clinocopathological data are summarized in Table 1 and a flow chart illustrating the datasets used in the explorative and validation phases is provided in Additional file 1. The study was approved by the regional Ethics Committee in Uppsala (2007/254) waiving the requirement for informed consent for the study.

**Table 1:** Clinicopathological data for the fresh frozen and paraffin-embedded MBC tumors, respectively

Clinicopathological characteristics	Fresh frozen tumors N (%)	Paraffin-embedded tumors N (%)
**Age at diagnosis**		
**Mean**	69	70
**Range**	42 to 93	23 to 98
**Tumor size**		
**T1**	18 (27)	93 (42)
**T2**	38 (58)	91 (41)
**N/A**	10 (15)	36 (16)
**Node status**		
**N0**	16 (24)	83 (38)
**N+**	37 (56)	78 (35)
**N/A**	13 (20)	59 (27)
**ER status**		
**Positive**	52 (79)	193 (88)
**Negative**	3 (5)	9 (4)
**N/A**	11 (17)	18 (8)
**PR status**		
**Positive**	46 (70)	160 (73)
**Negative**	9 (14)	41 (19)
**N/A**	11 (17)	19 (9)
***HER2 *status**		
**Positive**	2 (3)	18 (8)
**Negative**	35 (53)	157 (71)
**N/A**	29 (44)	45 (20)
***BRCA2 *mutation status**		
**Positive**	3 (5)	5 (2)
**Negative**	7 (11)	12 (5)
**N/A**	56 (85)	203 (92)
**Histology**		
**DCIS**	1 (2)	4 (2)
**Invasive cancer in combination with DCIS**	14 (21)	47 (21)
**Invasive cancer**	43 (65)	130 (59)
**N/A**	8 (12)	39 (18)
**NHG**		
**I**	2 (3)	15 (7)
**II**	17 (26)	98 (44)
**III**	19 (29)	85 (39)
**N/A**	28 (42)	22 (10)
**Metastases**		
**Yes**	16 (24)	46 (21)
**No**	39 (59)	123 (56)
**N/A**	11 (17)	51 (23)
**Follow-up time (years)**		
**Mean**	5.3	4.6
**Range**	0.20 to 15	0.04 to 15
**Adjuvant chemotherapy**		
**Yes**	6 (9)	21 (10)
**No**	51 (77)	159 (72)
**N/A**	9 (14)	40 (18)
**Adjuvant endocrine therapy**		
**Yes**	37 (56)	120 (55)
**No**	20 (30)	66 (30)
**N/A**	9 (14)	34 (15)
**Post-operative radiotherapy**		
**Yes**	30 (45)	85 (39)
**No**	28 (42)	96 (44)
**N/A**	8 (12)	39 (18)
**Surgery**		
**Mastectomy**	58 (88)	178 (81)
**Lumpectomy**	1 (2)	12 (5)
**No surgery**	0 (0)	2 (1)
**N/A**	7 (11)	28 (13)

### Gene expression (GEX) analysis

Tumor cellularity was determined on H&E stained sections and only tumors with high (> 70%) tumor cell content were included. Total RNA was extracted from fresh frozen tumors using the RNeasy Lipid Tissue Mini Kit (QIAGEN, Valencia, CA, USA) and RNA integrity was assessed on an Agilent 2100 Bioanalyzer (Agilent, Santa Clara, CA, USA). RNA quantification was performed using a NanoDrop ND-1000 (NanoDrop Products, Wilmington, DE, USA). Sixty-six samples with RIN values ≥ 7 were hybridized to Human HT-12 v3.0 Expression BeadChips (Illumina Inc, San Diego, CA, USA) in three batches at the SCIBLU Genomics Center at Lund University. Data normalization and management were performed using BioArray Software Environment (BASE) [23] and R [24]. Data were normalized using quantile normalization in BASE and were thereafter log2 transformed. To handle potential platform related biases, four samples each from hybridization batches one and two were re-hybridized in the third batch, resulting in a total of 74 experiments. A principal component analysis (PCA) was run and associations between principal components and technical and biological annotations were evaluated, whereupon a batch effect was detected as the main principal component (Additional file 2A). To correct for technical biases a supervised empirical Bayes method (ComBat) was thus applied [25]. PCA was then carried out on ComBat corrected data, whereupon no technical variation was found among the main principal components (Additional file 2B). The gene expression data have been published in NCBI's Gene Expression Omnibus (GEO) database (GSE31259) [26].

### Unsupervised discovery of MBC GEX subgroups

Probes with low signals (mean < 5.8 across all experiments) were filtered away and probes that varied the most across experiments were selected for use in unsupervised hierarchical clustering (HCL). Probes were mean centered across experiments. Pearson correlation distance and complete linkage were used for HCL. To assess the robustness of the initial HCL analysis, a multiscale bootstrap resampling was performed on the probes using the R-package Pvclust [27]. To further validate the stability of the clusters, resampling was performed on the samples to obtain 10,000 bootstrapped datasets. The co-clustering frequencies of sample pairs across the datasets were calculated. HCL was then carried out on the co-clustering frequencies and the dendrogram clusters were compared with the clusters from the initial HCL analysis as described [28]. When stable clusters were detected the procedure was repeated for each subcluster until no more stable clusters could be detected.

### Gene ontology

The Illumina probes were re-annotated using Re-annotation and Mapping for Oligonucleotide Array Technologies (ReMOAT) [29], and for the ontology studies only probes with good or perfect quality were used. A two-class unpaired significance analysis of microarray (SAM) was performed for MBC subgroups to identify differentially expressed genes, and the false discovery rate (FDR) 0 was used as a cut-off for significance. Up- and down-regulated genes were run separately in the database for annotation, visualization and integrated discovery (DAVID) v6.7 to identify possible enrichment of genes with specific biological themes separating the subgroups [30,31].

### Module signatures from FBC

Seven GEX modules associated with key biological processes in FBC (tumor invasion and metastasis, immune response, angiogenesis, apoptosis, proliferation, and ER and HER2 signaling, respectively) were used to discover biologically meaningful differences between MBC subgroups and to compare them with the intrinsic subgroups of FBC [21,32]. A score was computed for each module for all MBC samples as follows:

$$m.s.=\frac{\underset{i}{\sum }w_{i}x_{i}}{\underset{i}{\sum }\left|w_{i}\right|}$$

where *x_i _*is the expression of gene *i *in the module and *w_i _*is either +1 or -1 depending on the up- or down-regulation of each gene in the original FBC study [32]. The module scores were also calculated for a reference dataset representing all subgroups of FBC [22] as well as for an external dataset of MBC [15].

### Independent validation and comparison with FBC using external datasets

An external GEX dataset on custom made cDNA microarray slides containing 16,457 sequence-verified I.M.A.G.E. clones (Research Genetics, Invitrogen, Carlsbad, CA, USA) with 37 MBCs was downloaded from ArrayExpress (ID: E-TABM-810) [15]. Data quality assessment and normalization were performed in R [24]. Limma packages were used for background correction; a within-array method for data centering followed by a between-array method using quantile normalization. A normalized FBC dataset containing 359 FBC samples representing all intrinsic subgroups was downloaded from GEO (GSE22133) [22,26]. Samples in the validation sets were classified into MBC subgroups using nearest centroid classification. Centroids were calculated in our MBC data using the top 124 genes from the SAM analysis. Samples were classified based on to which centroid they showed the highest correlation and were unclassified if the correlation was < 0.2. We also performed nearest centroid classification for the intrinsic subgroups of FBC using the genes from Hu *et al*. on the MBC samples in our study as well as the MBC validation samples [21]. The Hu classifier relies on expression levels relative to the spectrum of FBC, that is, including both ER negative (ER-) and positive (ER+) samples. Since the vast majority of MBCs are ER+, we constructed an ER+ specific FBC subtype classifier. Briefly, ER+ samples were extracted from the FBC reference dataset and genes were mean centered across these samples. A SAM analysis was performed between the luminal A and B samples among these ER+ FBC samples, whereupon 300 significant genes with FDR = 0 were selected. Centroids were calculated for luminal A and B tumors separately using these 300 genes. Both MBC datasets were classified using these ER+ FBC luminal centroids. Finally, for the MBC validation dataset, the detection of stable subgroups was performed using the same unsupervised approach used for our dataset, and these subgroups were then compared with the subgroups from the centroid classification.

### Validation immunohistochemistry (IHC)

A tissue microarray (TMA) with two 1 mm cores from each of 220 MBC tumors was constructed as described [9]. Sections of 3 to 4 μm were cut, transferred to glass slides, dried at room temperature and then baked in a heat chamber for two hours at 60°C. The DAKO Envision horseradish peroxidase rabbit/mouse kit (DAKO, Glostrup, Denmark) and a Dakocytomation Autostainer (DAKO) were used for the staining procedure. A monoclonal mouse antibody to the polymorphic heavy chain of human MHC Class I (HC10, diluted in 1:1,000 in high pH), with preferential binding to HLA-B and HLA-C alleles and some HLA-A was generously provided by Prof. Dr. J. Neefjes [33,34], and the primary NAT1 antibody (diluted 1:1,000 in low pH) has been previously described [35,36]. The evaluation of NAT1 and HLA was performed by one reader (IJ) in a blinded manner. The intensity of the staining in the tumor cells was scored on a scale as: 0 (absent), 1 (weak), 2 (moderate) or 3 (strong). The percentage of positively stained tumor cells was scored as: 0 (< 5%), 1 (5 to 25%), 2 (26 to 50%), 3 (51 to 75%) or 4 (> 75%).

### Statistical analyses

All figures and statistical calculations were generated in R [24]. For the survival analyses the survival and survcomp packages were used with distant metastasis free survival (DMFS) as end-point. All *P*-values are two-sided.

## Results

### Discovery of two stable subgroups of MBC

**Figure 1:**
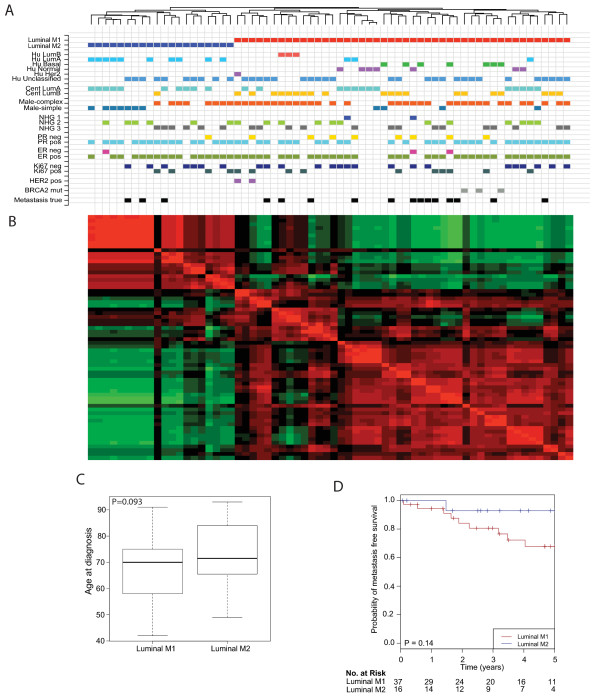
**Unsupervised hierarchical clustering (HCL) of male breast cancers based on 1,652 differential expressed genes**. **(A) **HCL revealed two stable subgroups, luminal M1 (right) and luminal M2 (left). Annotations with the prefix Hu indicate the result of the centroid classification based on the Hu genes [21]. Annotations with the prefix cent were derived from the centroid classification with the genes for ER+ luminal female breast cancer (FBC). NHG, Nottingham histological grade. **(B) **Unsupervised HCL based on co-clustering frequencies revealed two stable subgroups. Co-clustering frequencies close to 1 are red, close to 0 are green and equal to 0.5 are black. **(C) **Difference in age at diagnosis between the subgroups. **(D) **Kaplan-Meier survival analysis suggesting better distant metastasis free survival (DMFS) in the luminal M2 subgroup. The numbers below the plot indicate the number of patients at risk in each group at the given time points.

Unsupervised HCL on co-clustering frequencies revealed two stable subgroups of MBC (Figure 1A, B) as did Pvclust, where both clusters had an approximately unbiased (AU) probability of 94% [37]. The two subgroups could not be further subdivided into stable groups, perhaps due to the limited sample size. The larger subgroup (from hereon labeled luminal M1) contained 46/66 (70%) tumors and the smaller subgroup (labeled luminal M2) contained 20/66 (30%) tumors. The subgroups displayed different GEX patterns, as well as a tendency towards differences in age at diagnosis (Wilcoxon test, *P *= 0.093, Figure 1C). There was no difference in Nottingham histological grade (NHG, Fisher's Exact Test, *P *= 1.0) or tumor size (Wilcoxon test, *P *= 0.26) between the subgroups. The two subgroups correlated with the genomic subgroups (male-simple and male-complex, respectively; Figure 1A) that we previously defined based on genomic aberrations within the same patient cohort [14]; 89% of the luminal M1 tumors were classified as male-complex and 47% of the luminal M2 tumors were classified as male-simple (Fisher's exact test, *P *= 0.0079). Kaplan-Meier survival analysis indicated better survival in the luminal M2 subgroup; the difference in DMFS was, however, not statistically significant (*P *= 0.14, Figure 1D), most likely due to the limited number of events.

### Differences in key biological processes between MBC subgroups

**Figure 2:**
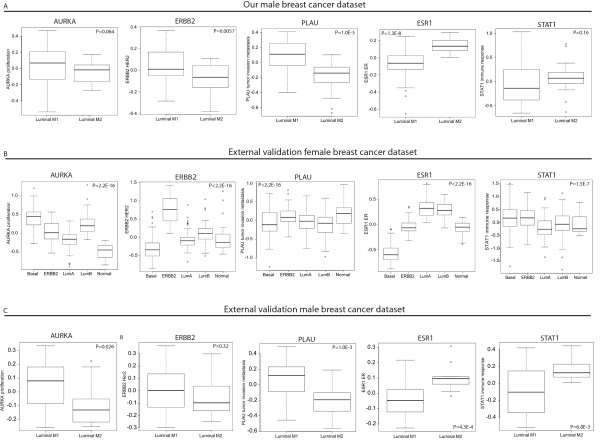
**Gene expression (GEX) modules associated with key biological processes**. The module scores of GEX modules representing key biological processes involved in FBC tumorigenesis [32] in the two subgroups of MBC **(A)**, in the intrinsic subgroups of FBC **(B)**, and in the MBC validation dataset **(C)**, respectively. Proliferation (Wilcoxon test, *P *= 0.064), HER2 (Wilcoxon test, *P *= 0.0057), tumor invasion and metastasis (Wilcoxon test, *P *= 1.0 × 10^-5^), ER (Wilcoxon test, *P *= 1.3 × 10^-8^) and immune response (Wilcoxon test, *P *= 0.16) displayed a significant or borderline significant difference between the two subgroups of MBC (A). The ANOVA test was used to calculate *P*-values **(B)**.

We investigated the expression of seven GEX modules, representing key biological processes involved in FBC tumorigenesis [32], in the respective subgroups to better characterize the biological foundation of MBC and the mechanisms underlying differences between the subgroups. Among the seven defined modules, proliferation (Wilcoxon test, *P *= 0.064), HER2 (Wilcoxon test, *P *= 0.0057), tumor invasion and metastasis (Wilcoxon test, *P *= 1.0 × 10^-5^), ER signaling (Wilcoxon test, *P *= 1.3 × 10^-8^) and immune response (Wilcoxon test, *P *= 0.16) displayed significant differences or a tendency towards differences between the two subgroups (Figure 2A). Luminal M1 tumors appeared more highly correlated to the tumor invasion and metastasis, proliferation and HER2 modules than luminal M2 tumors, further supporting the notion that luminal M1 tumors may be more aggressive than luminal M2 tumors. The expression of each of the modules in the intrinsic subgroups of FBC is shown in Figure 2B for comparison. Interestingly, neither the luminal M1 nor the luminal M2 subgroup of MBC displayed patterns of module scores resembling any of the intrinsic FBC subgroups. Of note, even though the majority of the MBC tumors were ER+ by IHC, the module score for ER signaling differed significantly between the subgroups (*P *= 1.3 × 10^-8^, Figure 2A). The ER+ subgroups of FBC (luminal A and B), on the other hand, both displayed very similar module scores for ER signaling, as expected (Figure 2B).

### Gene ontology indicates that luminal M1 tumors are more aggressive than luminal M2 tumors

A SAM analysis was performed, resulting in 544 up-regulated genes and 370 down-regulated genes in the luminal M2 compared to the luminal M1 subgroup (FDR = 0). The up- and down-regulated genes were uploaded separately into DAVID and some of the most relevant gene ontology (GO) terms associated with genes up-regulated in luminal M1 tumors included: cell migration, cell adhesion, angiogenesis, cell cycle, cell division and HOX genes (Additional file 3). Luminal M2 tumors, on the other hand, displayed up-regulation of genes associated with the GO term class I histocompatibility antigen, which is involved in the immune system (Additional file 4). Taken together, this suggests that luminal M1 tumors may be more aggressive than luminal M2 tumors, and may thus be associated with inferior outcome.

### Validation of the MBC subgroups with an independent external dataset

**Figure 3:**
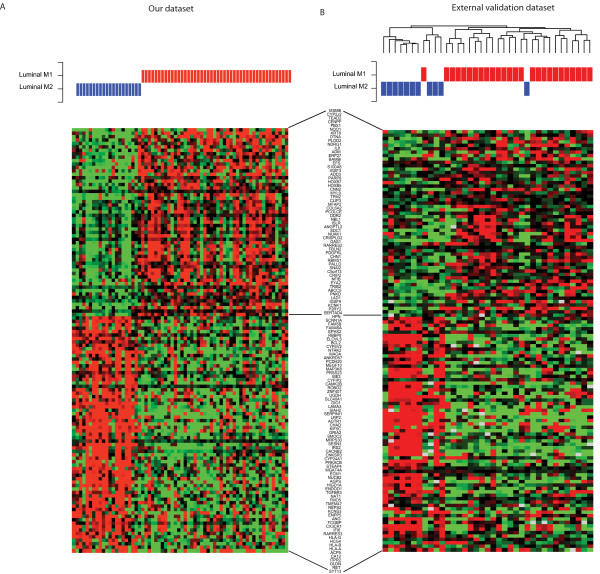
**Male breast cancer (MBC) subgroup specific genes**. Validation of two stable MBC subgroups in an external dataset. The heatmaps of the MBC subgroup-derived centroid genes revealed identical distribution frequencies and similar transcriptional profiles in our dataset **(A) **and the external validation dataset **(B)**. Red corresponds to up-regulation and green to down-regulation. The MBC sample order was derived from the unsupervised hierarchical clustering and the annotations are from the centroid classification with the MBC subgroup-derived centroid genes.

To further validate our discovery of two transcriptional subgroups we used an external MBC dataset consisting of 37 cases [15]. As a first step, we used MBC subgroup centroids from our data to classify the validation samples into the two subgroups, resulting in 19% unclassified samples (with a correlation cutoff > 0.2). When no correlation cutoffs were used, 26/37 (70%) tumors were classified as luminal M1 and 11/37 (30%) as luminal M2, identical to results for our dataset. In support of this finding, when an unsupervised approach was used to identify subgroups in the validation cohort, we found two stable subgroups comprising 11 and 26 tumors, respectively (Figure 3B). In the first subgroup 10/11 (91%) tumors were centroid classified as luminal M2, while in the second subgroup 25/26 (96%) tumors were centroid classified as luminal M1. To further support the validity of the identified subgroups, a comparison of the GEX patterns for the subgroup-derived centroid genes from our dataset and the validation dataset revealed highly similar patterns (Figure 3A, B). Finally, to additionally characterize the validation dataset, scores for the seven FBC GEX modules were calculated, revealing correlations between the respective modules and the two subgroups similar to our dataset (Figure 2A, C).

### The MBC subgroups differ from the intrinsic subgroups of FBC

In an effort to identify the degree of similarity between MBCs and the commonly used intrinsic subtypes of FBC [19,21], we applied a centroid-based approach based on the Hu genes to our MBC dataset. This classification left 55% of the samples unclassified. Taking into account that MBCs are generally ER+, we also classified the MBCs using ER+ FBC luminal subgroup centroids. Even using this approach, 36% of the samples remained unclassified. Conversely, when ER+ FBC samples were classified using the MBC subgroup centroids, 151 samples (63%) were unclassified. Interestingly, luminal M1 tumors showed significantly higher correlations to the HER2 and basal centroids (Wilcoxon test, *P *= 1.1 × 10^-3 ^and *P *= 6.8 × 10^-5^, respectively), while luminal M2 tumors were significantly higher correlated to both luminal A and B centroids (Wilcoxon test, *P *= 1.9 × 10^-5 ^and *P *= 0.018, respectively). Furthermore, pronounced differences in the GEX patterns were observed when the Hu genes and the ER+ FBC luminal subgroup centroid genes were compared across male and female breast cancers (Additional files 5 and 6). When the MBC tumors were clustered with the ER+ FBC subgroup centroid genes, the two MBC subgroups were mixed between the main clusters found (Additional file 7). These findings indicate that the MBC subgroups differ substantially from the intrinsic subgroups of FBC.

### NAT1 protein expression is prognostic for MBC

**Figure 4:**
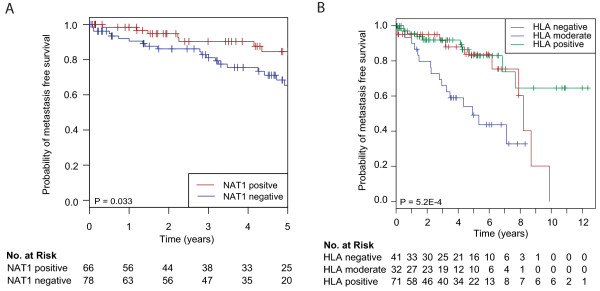
**Kaplan-Meier survival analyses**. Distant metastasis free survival of the 220 male breast cancer patients included in the TMA stratified by HLA expression **(A) **and NAT1 expression **(B)**, respectively. The numbers below the plots indicate the number of patients at risk in each group at the given time points.

Based on the finding that luminal M2 tumors displayed a higher correlation to the ER signaling module than luminal M1 tumors (Figure 2A), we investigated the protein expression of NAT1, one of the genes in this module, in 220 MBCs arranged in a TMA. *NAT1 *was also one of the top candidate genes from the SAM analysis, with a significantly higher expression in luminal M2 tumors compared to luminal M1 tumors. Tumors were considered positive for NAT1 if > 75% of the cancer cells showed cytoplasmic staining (Additional files 8A-C). Intense cytoplasmic staining was occasionally accompanied by nuclear staining, but this was not considered in the evaluation. A total of 113 (51%) tumors were NAT1 positive and 91 (41%) tumors were NAT1 negative (data were missing for 19 (8%) tumors). NAT1 protein expression correlated significantly to the mRNA levels (Spearman correlation 0.80, *P *= 1.7 × 10^-10^). A significant difference in the protein expression of NAT1 was seen between the subgroups, with more luminal M2 tumors being NAT1 positive compared to luminal M1 tumors (Fisher's exact test, *P *= 0.018). Furthermore, NAT1 negativity was associated with poor five-year DMFS in the whole cohort (hazard ratio (HR) 2.5 (95% CI 1.0 to 5.9) *P *= 0.033; Figure 4A). Importantly, the poor survival for patients with NAT1 negative tumors remained significant in a multivariate analysis when adjusting for node status, NHG, and tumor size (HR 2.8 (95% CI 1.0 to 7.2) *P *= 0.040; Table 2). To delineate whether NAT1 may predict response to endocrine therapy, we attempted to examine the association between NAT1 and DMFS separately in tamoxifen-treated patients; however, there were too few patients and events in the respective groups to perform this analysis (data not shown).

**Table 2:** Uni- and multi-variate analysis of five-year breast cancer specific survival

Variable	Univariate analysis			Multivariate analysis		
	
	HR	95% CI	*P*-value	HR	95% CI	*P*-value
**NAT1 positive**	2.5	1.0 to 5.9	0.033	2.8	1.0 to 7.2	0.040
**No *vs*. Yes**						
**Age**	1.4	0.33 to 5.8	0.66			
**< 45 *vs*. ≥ 45**						
**Tumor size**	2.9	1.3 to 6.6	0.008	2.4	0.82 to 6.8	0.11
**T2 *vs*. T1**						
**Node status**	3.3	1.4 to 7.9	0.005	3.1	1.1 to 8.3	0.029
**Pos *vs*. Neg**						
**NHG**	2.3	1.1 to 5.0	0.032	0.97	0.40 to 2.3	0.95
**3 *vs*. 1 to 2**						
**PR positive**	0.50	0.22 to 1.1	0.079			
**Yes *vs*. No**						

### The correlation between immune response and prognosis

Significant up-regulation of immune-related genes was observed in luminal M2 compared to luminal M1 tumors (Additional file 4), and a higher correlation to the immune module was also seen (Figure 2A, C). More specifically, class 1 HLA genes were strongly up-regulated in luminal M2 tumors. To explore the prognostic impact of immune-related genes we therefore assessed the protein levels of HLA in the extended cohort. Tumors were defined as positive, moderate or negative when > 50%, 5 to 50% or < 5% of the cancer cells were positive, respectively (Additional file 8D-F). Ninety-three (42%) tumors were positive, 51 (23%) displayed moderate staining and 58 (26%) were negative (data were missing for 21 (9%) tumors). A significant difference in the protein expression was observed, with the luminal M2 subgroup containing more HLA positive tumors than the luminal M1 subgroup (Fisher's exact test, *P *= 0.039). Most interestingly, HLA positivity was associated with significantly better DMFS than moderate HLA expression (HR 3.6 (95% CI 1.6 to 7.9) *P *= 0.002). The DMFS was similar in the HLA negative and the HLA positive groups during the beginning of the follow-up, but late events (> 8 years) among HLA negative cases nevertheless resulted in poor DMFS in the latter group, comparable to the moderate HLA group (Figure 4B).

## Discussion

While it is generally accepted that FBC is a heterogeneous disease both in terms of transcriptional profiles, genomic aberrations and survival [19-22], whether MBC can be classified into comprehensive subgroups associated with differences in clinicopathological variables has not yet been elucidated. The inferior outcome reported clearly indicates the requirement to better understand the pathobiology of MBC [9], and the need to stratify patients based on tumor characteristics, potentially in need of alternative treatment strategies.

In this study, we have performed GEX profiling on 66 MBC tumors to study the disease transcriptionally and to subclassify tumors based on mRNA profiles. We were thus able to stratify tumors into two stable subgroups, luminal M1 and luminal M2, respectively, associated with different biological features and clinicopathological characteristics. While additional subgroups may exist, the sample size was too small for further subdivisions. As evidenced from the SAM analysis, the two GEX subgroups displayed a large number of differentially expressed genes. Patients tended to be diagnosed at a younger age in the luminal M1 subgroup, but no differences in histological grade or tumor size were observed. Interestingly, the two subgroups correlated with the genomic subgroups (male-simple and male-complex, respectively) that we previously defined based on genomic aberrations within the same patient cohort [14]. Specifically, the luminal M2 subgroup described in the present study contained the majority of the male-simple tumors, while the luminal M1 subgroup harbored most of the male-complex tumors. There was a tendency towards better DMFS among patients with male-simple tumors with few genomic aberrations compared to patients with male-complex tumors with highly rearranged genomes and survival comparable to luminal B FBCs. Male-simple tumors were less frequently aneuploid, displayed a lower fraction of genome altered as well as lower S-phase fractions [14], further supporting better outcome among patients with luminal M2 (male-simple) *vs*. luminal M1 (male-complex) tumors.

To better understand the biological differences between the two subgroups, we performed an ontology analysis, whereupon up-regulation of genes involved in cell migration, cell adhesion, angiogenesis, cell cycle, cell division and HOX genes was identified among luminal M1 tumors. Two of the well-known hallmarks of cancer development are activation of invasion and metastasis and induction of angiogenesis, respectively [38]. Up-regulation of genes involved in these processes in the luminal M1 subgroup indicates that these tumors may have a more aggressive phenotype than luminal M2 tumors. HOX genes are DNA binding factors involved in the transcriptional regulation of many key development factors, and de-regulated expression of HOX genes has been found to be involved in carcinogenesis and metastasis in many different cancer forms, including breast cancer [39]. In the present study, HOXB7, which has been shown to be involved in epithelial-mesenchymal transition, migration and invasion in FBC [40], was significantly up-regulated in luminal M1 tumors compared to luminal M2 tumors. This finding also corresponds to the higher frequency of distant metastases in the former group.

An additional hallmark of cancer pertains to activation of the immune system, specifically by T and B lymphocytes, macrophages and natural killer cells [38]. To this end, luminal M2 tumors displayed high expression of genes associated with class I histocompatibility antigens, which are involved in regulating the immune response, further supporting a more favorable outcome among luminal M2 tumors. A Kaplan-Meier survival analysis indeed suggested better DMFS among luminal M2 tumors, lending additional support to this notion.

To further understand the biological differences between the MBC subgroups, we investigated seven key biological processes associated with differences in survival among the intrinsic subtypes of FBC by calculating scores for each module in the two MBC subgroups. The luminal M2 subgroup demonstrated higher scores for the immune response and ER modules, while luminal M1 tumors displayed higher scores for the tumor invasion and metastasis, proliferation and HER2 modules, again indicating differences in tumor aggressiveness between the subgroups. A surprising finding was the low score for the ER module among luminal M1 tumors, despite almost all MBC tumors in the present study being ER+. In FBC, only ER- tumors display a low ER module score, suggesting that luminal M1 MBC tumors, although positive by IHC, in fact differ from ER+ FBCs. Luminal M2 MBC tumors also appear to differ from the FBC intrinsic subgroups, as the correlations between module scores and ER+ FBC intrinsic subgroups were not recapitulated. While some similarities to both luminal A and B subgroups were observed among luminal M2 tumors, a high module score for immune response was also seen, a feature only associated with the HER2 and basal intrinsic subtypes of FBC. These findings underscore the difficulty in capturing the complexity of molecular alterations associated with MBC subtypes using single protein markers. To date, only two other studies have attempted to subclassify MBC into the major FBC intrinsic subtypes. Applying IHC and the commonly used protein markers for FBC subtyping, approximately 80% of the tumors were classified as luminal A and approximately 20% as luminal B [41,42]. The discrepancy with our findings is probably due to the inability of a small number of protein markers to fully capture the differences in transcriptional profiles between subtypes. This is illustrated by, for example, luminal M1 tumors being ER+ by IHC, while displaying less active ER signaling, more similar to ER- FBC.

Several studies of FBC have underlined the importance of regulation of the immune system in ER- and HER2 positive (HER2+) tumors [43-45]. Teschendorf *et al*. identified an immune response related seven-gene signature in ER- tumors correlated to risk of distant metastases [43,44]. Further, Staaf *et al*. defined a predictor prognostic of outcome for HER2+ FBC tumors that included genes associated with immune response, tumor invasion and metastasis. The better prognosis group displayed up-regulation of the immune response and low invasive ability. Of interest, this predictor also performed well in ER- FBC, but not in ER+/HER2- FBC [45]. Given the correlation to the immune module in the luminal M2 subgroup of ER+ MBCs, and the lack of association with the ER signaling module in the luminal M1 subgroup, these findings indicate that the two subgroups of MBC described herein may constitute two new subgroups of breast cancer, with unique biological and clinical features, occurring only in males. Hypothetically, these patients may require novel treatment strategies. Specifically, despite the majority of tumors in the luminal M1 subgroup being ER+ they had a low ER signaling module score, suggesting that the ER pathway may not be active. A recent comprehensive study of steroid hormone receptors in breast cancer revealed gender specific differences, suggesting differential hormonal dependency [46]. Hence, whether these MBC patients respond to endocrine therapy like tamoxifen may be questioned, and needs to be further investigated in prospective randomized studies. Intriguingly, a recent study implicated HOXB7, one of the genes up-regulated in luminal M1 MBC tumors, in rendering FBC cells resistant to tamoxifen. In addition, high expression of HOXB7 in tamoxifen treated FBC patients correlated with poor disease free survival [47].

Importantly, we were able to validate the MBC subgroups in an independent dataset [15]; the centroid classification in this dataset resulted in the same distribution into the two subgroups as in our dataset, and unsupervised clustering revealed two stable subgroups with similar characteristics as those found in our dataset. Specifically, the GEX patterns of the centroid genes were similar and the module scores showed the same trends in the validation dataset, indicating similar associations with biological processes. Unfortunately, however, no information on outcome was available from that study.

A comparison between the MBC subgroups and a dataset representing all intrinsic subgroups of FBC revealed pronounced differences; 55% and 36% of the MBC samples were unclassified when applying the Hu gene centroid and FBC ER+ luminal gene centroid classifications, respectively. The fraction of unclassified tumors among FBCs has previously been reported to be 0 to 20% [48]. The GEX patterns based on these genes also differed between male and female breast tumors, further indicating that the subgroups of MBC identified herein are not represented by the intrinsic subgroups of FBC. When our MBC samples were clustered based on the ER+ FBC luminal centroid genes, the two MBC subgroups were intermixed between the clusters. In support of this finding, our previous study of genomic profiles in MBC also revealed that the two MBC subgroups largely differed from genomic subgroups described in FBC [14]. Although some of the centroid genes in ER+ luminal FBC may differ between the MBC subgroups, they are clearly not the most dominant feature.

To further characterize the subgroups and investigate the clinical relevance of our findings, we investigated NAT1 and HLA protein levels in a series of 220 MBCs, as differences in mRNA levels of the corresponding genes were found between the subgroups. A significantly worse DMFS was observed for the moderate HLA group compared with the positive HLA group. Curiously, however, the HLA negative group initially had a prognosis similar to the HLA positive group, but late recurrences resulted in worse long-time survival. Due to the fairly small sample size and the fact that not many patients remained alive after eight years, this finding needs to be interpreted cautiously and more studies are needed to validate these findings. Interestingly, women with node-negative breast cancer displaying a mixed HLA class I expression pattern had a worse prognosis according to a study by Gudmundsdóttir *et al. *[49]. Tumors with high expression of HLA class I can be recognized by T lymphocytes of the specific immune system. On the other hand, tumors lacking expression of HLA class I may be targeted by NK cells of the non-specific immune system [50,51]. Tumors with a mixed expression of HLA class I may thus hypothetically be able to avoid the specific immune system by displaying too few antigens, while evading the non-specific immune system by inhibiting NK cell activity [49,51].

Several studies have reported higher expression of NAT1 on both protein and mRNA levels in ER+ FBC compared to ER- FBC [20,52-54], and high expression of NAT1 has been shown to correlate with better outcome among ER+ FBCs [55,56]. The NAT1 antibody used in the present study has been used over the past 20 years with consistent results on cellular localization [35,36,55], and has been demonstrated to be uniquely specific following Western blot analysis [52]. However, there has also been an indication of a nuclear location [57], which has been linked to turnover of the NAT1 protein. The precise role of the small proportion of nuclear staining of NAT1 has not been established, but may well be related to protein turnover and gene regulation. Patients whose tumors were positive for NAT1 displayed a significantly better prognosis than those with NAT1 negative tumors in the present study, a finding that remained significant in a multivariate analysis. ER status was not included in the multivariate analysis, because only seven tumors were ER-. It has, however, been shown that ER status provides independent prognostic information in MBC [6]. Luminal M2 tumors displayed higher NAT1 levels compared to luminal M1 tumors, thus further supporting an association between the luminal M2 subgroup and better outcome. Bièche *et al*. found high NAT1 to be predictive of response to tamoxifen in women with ER+ breast cancer. In general, altered tamoxifen metabolism and bioavailability may contribute to tamoxifen resistance, and the xenobiotic-metabolizing enzyme NAT1 may be part of this explanation [56]. Unfortunately, we had too few cases to be able to detect any association between NAT1 and survival among only tamoxifen treated patients, but this finding warrants further investigation and suggests that the low expression of NAT1 among luminal M1 tumors may lead to tamoxifen resistance, and that these patients may hence require alternative treatment approaches.

## Conclusions

We have detected two unique and stable subgroups of MBC with differences in tumor biological features and outcome, luminal M1 tumors being more aggressive and associated with worse prognosis, while luminal M2 tumors, on the other hand, displayed up-regulated immune response and activated ER signaling, generally favorable features. Importantly, both MBC subgroups differed from the established intrinsic subgroups of FBC, indicating that they constitute two novel subgroups of breast cancer occurring only in males. Consequently, men diagnosed with breast cancer may require other management and treatment strategies than women. Finally, we identified NAT1 as a possible prognostic biomarker for MBC, as suggested by NAT1 positivity corresponding to better outcome.

## Abbreviations

aCGH: array-based comparative genomic hybridization; AU: approximately unbiased; BASE: BioArray Software Environment; CGH: comparative genomic hybridization; DAVID: The Database for Annotation; Visualization and Integrated Discovery; DMFS: distant metastasis free survival; ER: estrogen receptor; FBC: female breast cancer; FDR: false discovery rate; GEO: Gene Expression Omnibus; GEX: gene expression; GO: gene ontology; HCL: hierarchical clustering; HER2: human epidermal growth factor receptor 2; HLA: class 1 human leukocyte antigen; HR: hazard ratio; MBC: male breast cancer; NAT1: *N*-acetyl transferase-1; NHG: Nottingham Histological Grade; PCA: principal component analysis; PR: progesterone receptor; ReMOAT: Re-annotation and Mapping for Oligonucleotide Array Technologies; SAM: significance analysis of microarray; TMA: tissue microarray.

## Competing interests

The authors declare they have no competing financial interests.

## Authors' contributions

IH, MLF and IJ were responsible for study design. IJ and PB carried out microarray experiments. CN, MLF, LL and HO contributed samples and patient information. IJ and CN performed immunohistochemistry evaluations. ST performed histological grading. ES provided the NAT antibody. IJ performed bioinformatics and statistical analyses with support from MR and ML. IJ, MR and IH interpreted the data. IJ and IH drafted the manuscript. All authors read and approved the final manuscript.

## Supplementary Material

Additional file 1 **Flow of datasets in the explorative and validation phases**. Click here for file

Additional file 2 **Principal component analyses (PCA)**. A PCA was performed and associations between principal components and technical and biological annotations were evaluated, whereupon a platform-specific bias was detected in the main principal component **(A)**. After adjustment using ComBat [25], no technical variation was found among the main principal components **(B)**. *NHG, Nottingham histological grade. Click here for file

Additional file 3 **Enrichment in biological process GO terms of the genes up-regulated in luminal M1 tumors *vs*. luminal M2 tumors**. Click here for file

Additional file 4 **Enrichment in biological process GO terms of the genes up-regulated in luminal M2 tumors *vs*. luminal M1 tumors**. Click here for file

Additional file 5 **Heatmaps of the intrinsic genes for female breast cancer (FBC)**. Expression of the intrinsic genes according to Hu *et al*. (21) in the FBC validation dataset **(A) **and our male breast cancer (MBC) dataset **(B)**. Red corresponds to up-regulation and green to down-regulation. Click here for file

Additional file 6 **Heatmaps of ER positive luminal female breast cancer centroid genes**. Expression of the ER+ luminal FBC centroid genes in the FBC validation dataset **(A) **and our MBC dataset **(B)**. Red corresponds to up-regulation and green to down-regulation. Click here for file

Additional file 7 **Hierarchical clustering (HCL) of male breast cancer (MBC) with ER positive luminal female breast cancer (FBC) centroid genes**. Unsupervised HCL of our MBC dataset based on the ER+ FBC centroid genes. The annotations indicate the two MBC subgroups. Click here for file

Additional file 8 **Immunohistochemical detection of NAT1 (A-C) and HLA (D-F) in paraffin-embedded male breast cancer tumors using a 20x objective**. (A) A NAT1 positive tumor with > 75% positive cancer cells. (B-C) Two NAT1 negative tumors with < = 75% positive cancer cells. (D) An HLA positive tumor with > 50% positive cancer cells. (E) An HLA moderate tumor with 5 to 50% positive cancer cells. (F) An HLA negative tumor with < 5% positive cancer cells. Click here for file
