# Supplementary figures and images for: Gene expression profiling of primary male breast cancers reveals two unique subgroups and identifies N-acetyltransferase-1 (NAT1) as a novel prognostic biomarker
Source: Breast Cancer Res. 2012 Feb 14;14(1):R31. doi: 10.1186/bcr3116 (PMC3496149; doi:10.1186/bcr3116)

## Additional 1

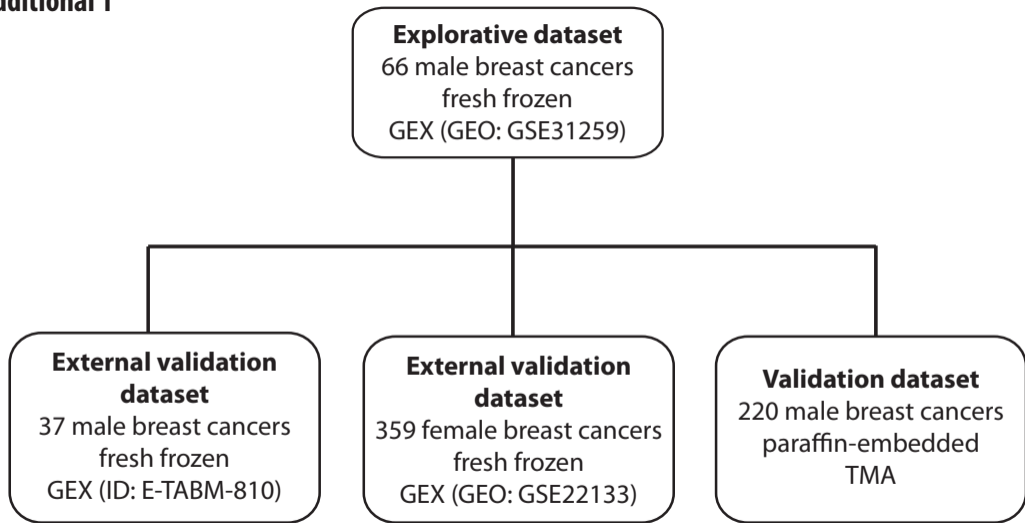

Supplement: Additional file 1 — Flow of datasets in the explorative and validation phases. [file bcr3116-S1.PDF]

## Additional 2

### A Quantile normalization

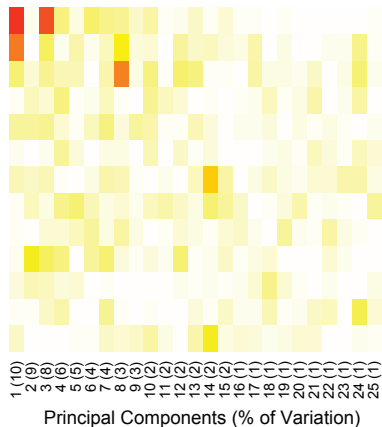

### B Quantile normalization and Combat

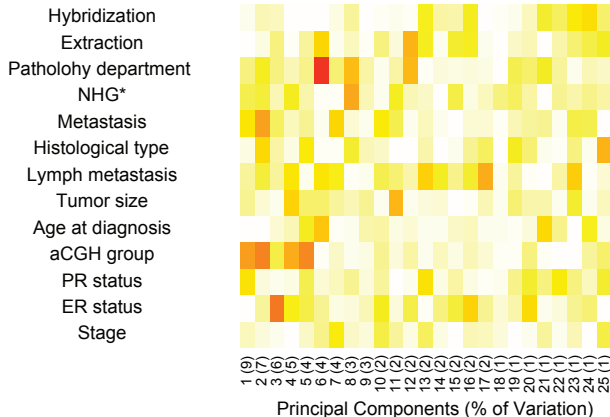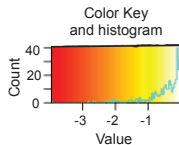

Supplement: Additional file 2 — Principal component analyses (PCA). A PCA was performed and associations between principal components and technical and biological annotations were evaluated, whereupon a platform-specific bias was detected in the main principal component (A). After adjustment using ComBat [25], no technical variation was found among the main principal components (B). *NHG, Nottingham histological grade. [file bcr3116-S2.PDF]

# Additional 5

A

Luminal A  
Luminal B  
HER2  
Normal  
Basal  
Unclassified

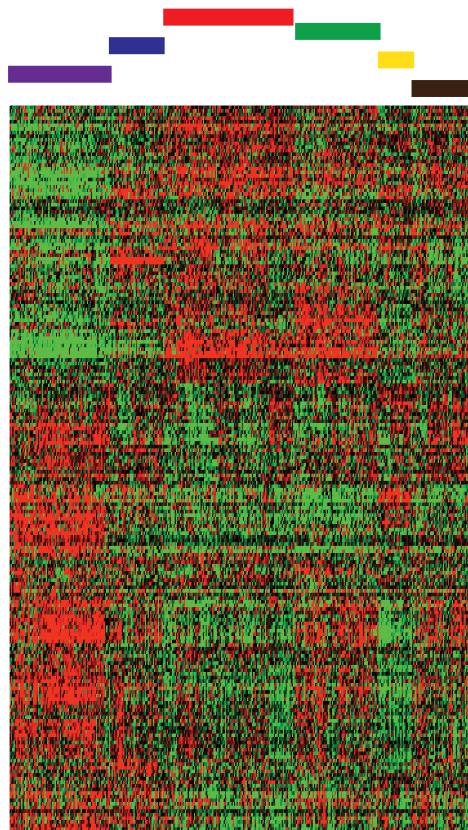

B

Luminal M1  
Luminal M2

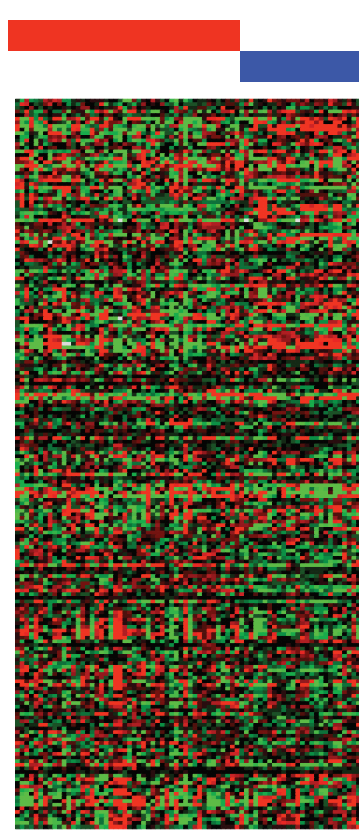

Supplement: Additional file 5 — Heatmaps of the intrinsic genes for female breast cancer (FBC). Expression of the intrinsic genes according to Hu et al. (21) in the FBC validation dataset (A) and our male breast cancer (MBC) dataset (B). Red corresponds to up-regulation and green to down-regulation. [file bcr3116-S5.PDF]

## Additional 6

A

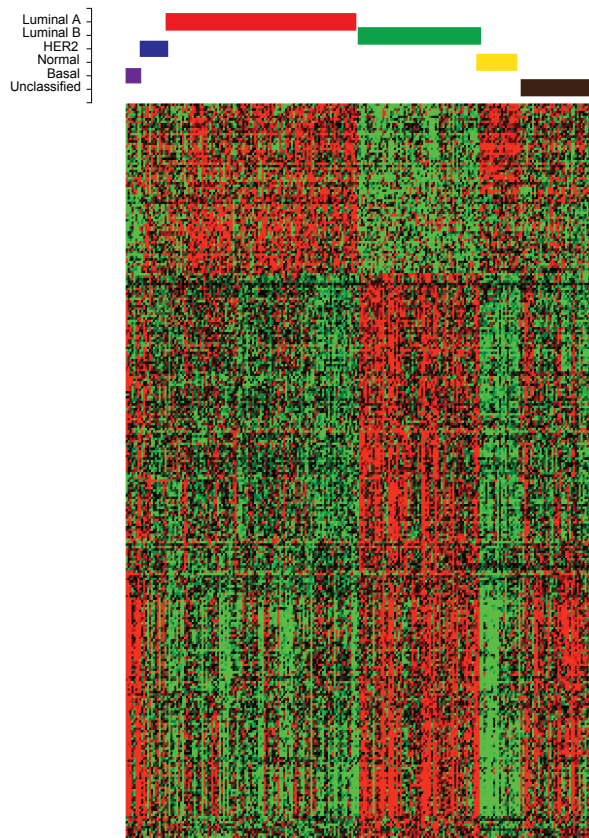

B

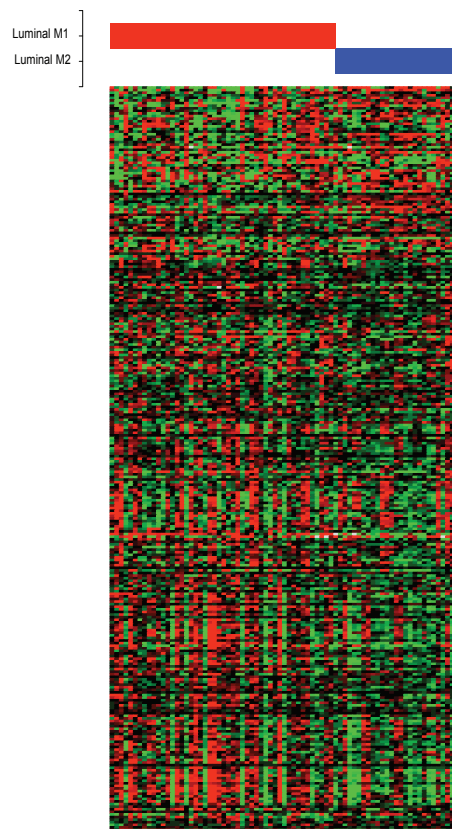

Supplement: Additional file 6 — Heatmaps of ER positive luminal female breast cancer centroid genes. Expression of the ER+ luminal FBC centroid genes in the FBC validation dataset (A) and our MBC dataset (B). Red corresponds to up-regulation and green to down-regulation. [file bcr3116-S6.PDF]

Additional 7

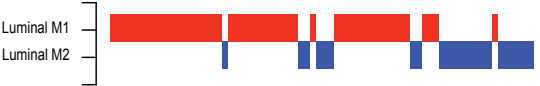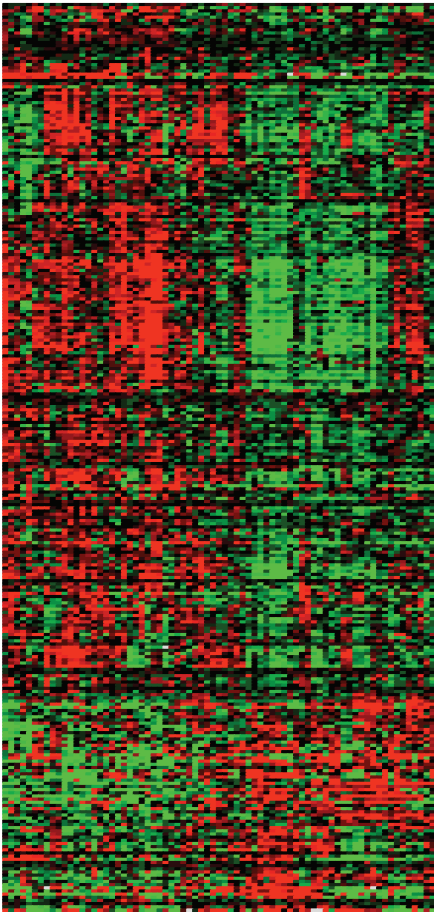

Supplement: Additional file 7 — Hierarchical clustering (HCL) of male breast cancer (MBC) with ER positive luminal female breast cancer (FBC) centroid genes. Unsupervised HCL of our MBC dataset based on the ER+ FBC centroid genes. The annotations indicate the two MBC subgroups. [file bcr3116-S7.PDF]

## Additional 8

### NAT1

---

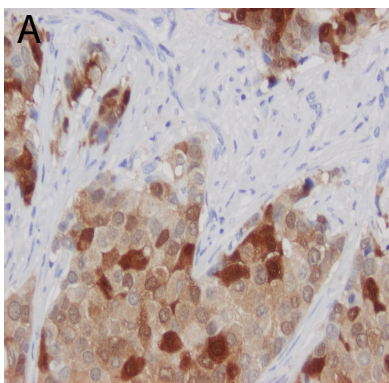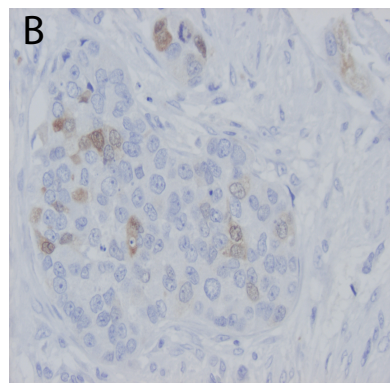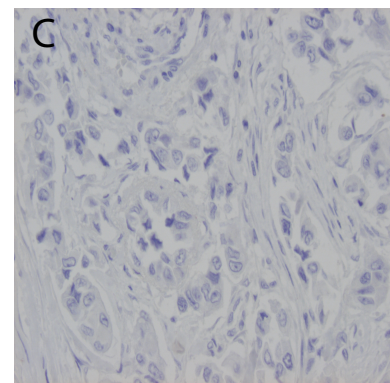

### HLA

---

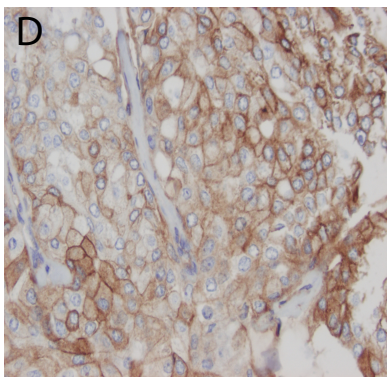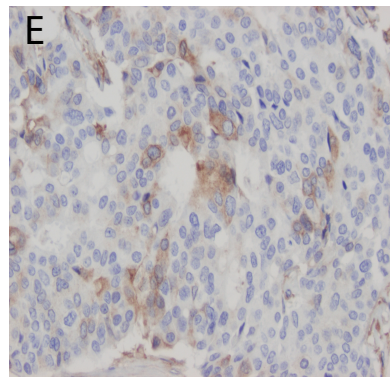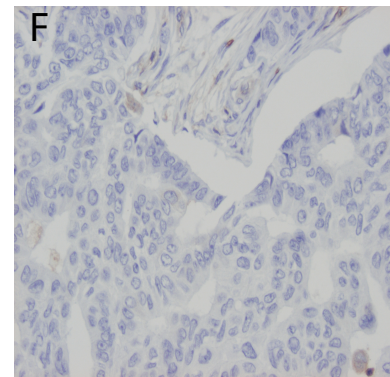

Supplement: Additional file 8 — Immunohistochemical detection of NAT1 (A-C) and HLA (D-F) in paraffin-embedded male breast cancer tumors using a 20x objective. (A) A NAT1 positive tumor with > 75% positive cancer cells. (B-C) Two NAT1 negative tumors with < = 75% positive cancer cells. (D) An HLA positive tumor with > 50% positive cancer cells. (E) An HLA moderate tumor with 5 to 50% positive cancer cells. (F) An HLA negative tumor with < 5% positive cancer cells. [file bcr3116-S8.PDF]
